# Fournier gangrene due to Rhizobium Radiobacter

**DOI:** 10.12669/pjms.344.14489

**Published:** 2018

**Authors:** Volkan Sen, Pinar Sen, Mehmet Oguz Sahin

**Affiliations:** 1 Volkan Sen, M.D., FEBU. Department of Urology, Manisa State Hospital, Manisa, Turkey; 2 Pinar Sen, M.D. Department of Infectious Diseases and Clinical Microbiology, Manisa Saruhanli State Hospital, Manisa, Turkey; 3 Mehmet Oguz Sahin, M.D. Department of Urology, Manisa State Hospital, Manisa, Turkey

**Keywords:** *Agrobacterium*, Fournier gangrene, Immunocompromised, Infection, Rhizobium radiobacter

## Abstract

Fournier’s gangrene (FG) is a life-threatening, rapidly progressing acute necrotizing fasciitis of perianal, genitourinary and perineal areas. The most common isolated pathogens are *E.coli*, *Streptococcus* and *Enterococcus* in tissue and abscess cultures. However we present the first Rhizobium radiobacter FG in this case. A 47-year-old man presented with black necrotizing area with malodorous pus drainage in the bottom of the right hemiscrotum. Intravenous imipenem and Clindamycin were started prophylactically; all necrotizing tissues were debrided and right hemiscrotectomy was performed. Rhizobium radiobacter was isolated from tissue and abscess cultures. After successful treatment patient was discharged posteroperative 18^th^ day. The combination therapy of early and totally surgical debridement of necrotic tissues and appropriate antibiotic use are adequate to control Rhizobium radiobacter infection in FG.

## INTRODUCTION

Fournier’s gangrene (FG) is a life-threatening, rapidly progressing acute necrotizing fasciitis of perianal, genitourinary and perineal areas and was first described by Fournier.^1^,^2^ Mortality rates of FG were reported between 3-60% in previous studies.^2^,^3^ Early surgical debridement of all necrotic tissues, broad spectrum antibiotics and supportive care are the standard treatment modalities of FG. FG is a generally polimicrobial disease and the most common isolated pathogens are *E.coli*, *Streptococcus* and *Enterococcus* in tissue cultures.^2^,^4^ However; to the best of our knowledge Rhizobium radiobacter, a soil-based pathogen has not been reported as a cause of FG in literature. In this case we wanted to report the first Rhizobium radiobacter FG in the literature.

## CASE REPORT

**Fig.1:**
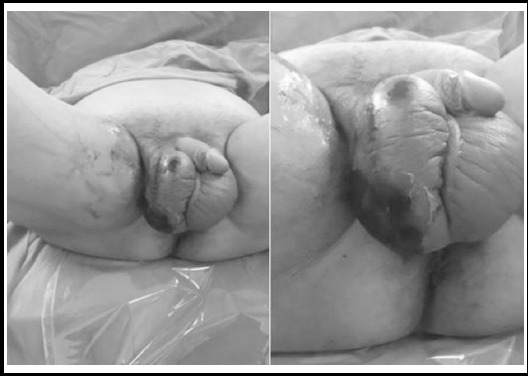
Massive edema in both of two hemiscrotum and black necrotizing area with malodorous pus drainage in the bottom of the right hemiscrotum.

**Fig.2:**
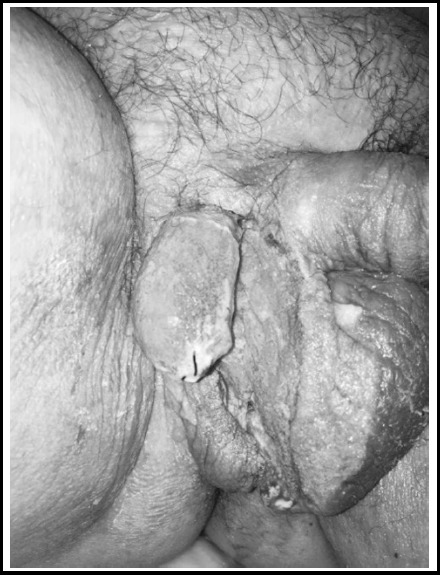
Postoperative 2^nd^ day, right testis had a normal blood supply appearance.

A 47-year-old man presented with a 10-day history of right scrotal pain, swelling and erythema. He had malodorous drainage from right scrotum for two days. He had no diabetes mellitus (DM), hypertension or any other co-morbid diseases; also there were no any risk factors including drug-use, immunodeficiency, genito-urinary or anorectal trauma and infection in his medical history. Massive edema in both of two hemiscrotum and black necrotizing area with malodorous pus drainage in the bottom of the right hemiscrotum was detected in his physical examination (Fig.1). His anorectal examination was normal. Laboratory analysis revealed as a serum creatinin 0.9 mg/dl, hemoglobin 14.9 g/dl, glucose 486 mg/dL, CRP 156 mg/L, WBC14.5x10^6^ cells/mL, sodium 132 mmol/L. There was no infection sign in bilateral testis, but their sizes were found smaller in scrotal ultrasound (right testis 25x20 mm, left testis 20x20 mm). Intravenous crystallized insulin therapy was given for decreasing serum glucose levels and intravenous imipenem 4x500 mg and Clindamycin 4x600 mg were started prophylactically to the patient according to the infectious disease consultation. All necrotizing tissues were debrided in right scrotum. Right hemiscrotectomy was performed and right testis had a normal blood supply appearance in operation (Fig.2). Open wound dressing with the nitrofurazone and rifamycin was performed in first three days after operation. The vacuum-assisted closure technique (VAC) (a technique that keeps the wound environment under sterile condition and decreases the frequency of changing protective covers of wound) was performed to the patient in postoperative 3^rd^ day to postoperative 15^th^ day. VAC dressing makes its function by creating mechanical stress with negative pressure of the vacuum system; wound edges diminish, granulation formation accelerates, cellular proliferation and neoangiogenesis increases.

Pathological result revealed as Fournier gangrene and Rhizobium radiobacter was isolated from both of the tissue and abscess cultures. Rhizobium radiobacter was only resistant to trimethoprim sulfamethoxazole and sensitive to ceftazidime, cefepime, imipenem, meropenem, ertapenem, amikacin, gentamycin and tetracycline; therefore same antibiotics were continued to the patient. Control tissue culture was done at postoperative 13^th^ day and by the results revealed as negative wound site was sutured primarily at postoperative 15^th^ day. Patient was discharged at postoperative 18^th^ day with the suggestion of endocrinology consultation for a new diagnosed diabetes mellitus. Patient had come to third month control, he had no symptoms and his scrotum was normal.

## DISCUSSION

FG is a life-threatening and usually a polimicrobial disease. The most common isolated pathogens are *E.coli*, streptococcus and *Enterococcus* in tissue and abscess cultures however we present the first Rhizobium radiobacter FG in this case. Rhizobium radiobacter was firstly isolated from human clinical specimens by Lautrop in 1967.^5^ Rhizobium radiobacter (Agrobacterium radiobacter) is a ubiquitous, aerobic, motile, oxidase positive, non-spore forming soil organism.^6^ It is usually known as a plant pathogen however can lead infections in human very rarely especially in immunocompromised patients.^7^ The type of contamination in patients with Rhizobium radiobacter infection remains hypothetical. Most of the patients in reports had outdoor activities such as gardening or golfing that may have exposed them to soil bacteria.^8^,^9^ Similarly with literature our patient had a story of gardening for a year.

Most of the Rhizobium radiobacter infections reported in literature were associated with contamination of intravenous fluid in patients with immunocompromised disease, including malignancies or HIV infection, or a history of indwelling central venous catheters.^7^,^10^ Dhatariya et al. reported that diabetes mellitus was also a risk factor for Rhizobium radiobacter infections.^6^ They pointed that poorly controlled diabetes can represent a form of immunocompromised, with infections being more common in those with diabetes and it is known that hyperglycaemia has negative effects on wound healing steps including inflammation, cell injury, apoptosis, endothelial function, the coagulation cascade and platelet aggregation.^6^ The diagnosis of diabetes mellitus in our patient was made preoperatively and patient was not being aware of DM before.

There is no standard therapy for Rhizobium radiobacter infections due to its low virulence and incidence. In previous reports local antimicrobial susceptibility tests showed that Rhizobium radiobacter was most commonly sensitive to 3rd generation cephalosporins, aminoglycosides, fluroquinolones and carbapenems.^6^,^10^ Rhizobium radiobacter was only resistant to trimethoprim sulfamethoxazole in susceptibility report of tissue and abscess culture of our patient, so intravenous imipenem and Clindamycin started with prophylactically for FG and were continued until the control tissue culture had become negative.

## CONCLUSION

Rhizobium radiobacter is an opportunistic soil-based bacteria leads to infection generally in immunocompromised patients. The combination therapy of early and totally surgical debridement of necrotic tissues and appropriate antibiotic use are adequate to control Rhizobium radiobacter infection in FG. .

### Authors’ Contribution

**VS, PS, MOS:** Conceived, designed and did statistical analysis & editing of manuscript.

**VS, PS:** Did data collection and manuscript writing.

**VS, PS, MOS:** Takes the responsibility and is accountable for all aspects of the work in ensuring that questions related to the accuracy or integrity of any part of the work are appropriately investigated and resolved.
